# The Value of Surveillance Imaging of Oral Squamous Cell Carcinoma

**DOI:** 10.3390/cancers16010207

**Published:** 2024-01-01

**Authors:** Oliver Bissinger, Anne Von den Hoff, Elisabeth Maier, Katharina Theresa Obermeier, Herbert Stimmer, Andreas Kolk, Klaus-Dietrich Wolff, Carolin Götz

**Affiliations:** 1 Department of Oral and Maxillofacial Surgery, Medical University of Innsbruck, Anichstraße 35, 6020 Innsbruck, Austriacarolin.goetz1@googlemail.com (C.G.); 2 Department of Oral and Maxillofacial Surgery, School of Medicine, Technical University of Munich, Klinikum Rechts der Isar, Ismaninger Str. 22, 81675 Munich, Germany; 3 Department of Oral and Maxillofacial Surgery and Facial Plastic Surgery, University Hospital LMU Munich, Lindwurmstraße 2A, 80337 Munich, Germany; 4 Department of Radiology, School of Medicine, Technical University of Munich, Klinikum Rechts der Isar, Ismaninger Str. 22, 81675 Munich, Germany

**Keywords:** oral squamous cell carcinoma, CT/NMRI, follow-up, surveillance imaging, false positives

## Abstract

**Simple Summary:**

Oral squamous cell carcinoma is the most common oral carcinoma worldwide. Despite medical improvements and applied research, the 5-year- overall survival rate amounts to ca 51 percent. Early recurrence diagnosis is a key driver for increasing the cure rate which underlines the importance of this study. The study aims to show how surveillance imaging of OSCC patients might be influenced by factors such as radiotherapy treatment, contrast enhancement, and the type of imaging (CT, NMRI). Further analysis is needed to determine if there is any difference between patients showing clinical and radiological patterns or only radiological patterns and if there is any connection between the histopathological result and those influence factors to improve the value of surveillance imaging in follow-up treatment and thus the cure rate.

**Abstract:**

The evaluation of surveillance imaging of OSCC patients is a difficult task physicians have to face daily. Multiple patients experience a recurrence of this disease, which underlines the importance of regular patient monitoring programs. Our study analysed the value of surveillance imaging, such as computed tomography (CT) and nuclear magnetic resonance imaging (NMRI), as a patient monitoring programme and its effectiveness in achieving improvement in early recurrence detection. The study comprised 125 patients, out of which 56 (*n* = 56) showed radiological and 69 (*n* = 69) showed clinical and radiological conspicuous patterns in domestic follow-ups, respectively. The use of CT and NMRI showed a significant dependence on the histological result (*p* = 0.03). However, the different groups showed no significant dependence on the histological result (*p* = 0.96). The distribution of the histological biopsies, which were taken due to radiological changes, were prone to wrong positive diagnoses (false positives) in 71 percent. To conclude, imaging modalities should be chosen for each patient individually to reduce false positives, improve the early detection of recurrence, and increase the cure rate.

## 1. Introduction

Oral squamous cell carcinoma (OSCC) is one of the most prevalent and grave oral tumours worldwide [1]. Due to its many early recurrences, the recurrence rate for OSCC is between 25–30%, with around 76% of recurrences occurring in the first two years, especially in the oral tongue area; follow-up routines should not be underestimated [2]. Early detection of recurrences ensures better physical and psychological treatment conditions for patients [3]. The aim should be to detect recurrences while they are still asymptomatically [4]. This underlines the importance of the value of surveillance imaging. With high imaging quality, early detection of asymptomatic recurrences and the right diagnosis could be achieved [5]. Follow-up routines generally differ among physicians and last approximately five years. It is recommended to schedule more frequent follow-up meetings in the first two years and subsequently decrease the frequency for years three to five [3]. However, it is also recommended that follow-up periods over three years should be discussed with the patients because recurrences primarily occur within the first three years [6]. In terms of follow-up frequency, the German S3-guideline for diagnostics and therapy of oral cavity carcinomas recommends a three-month interval for the first two years and a six-month interval for years three to five [7]. The use of surveillance imaging (CT/NMRI) during follow-up treatments should be applied semi-annually for the first two years and annually for years three to five. The most common imaging method is CT due to its short duration and cost-effectiveness [8]. Unfortunately, NMRI is underestimated as surveillance imaging, although it has a higher sensitivity for muscle infiltrations and a better outcome regarding artefacts [9]. Furthermore, a closer examination and more stringent indication should be intended to achieve a patient’s well-being because patients’ main fear is recurrence [10]. Furthermore, physicians are prone to evaluate changes in imaging as recurrences, which stem from the consequences that patients and physicians have to face afterwards: False positives result in an unnecessary operation, while false negatives result in a recurrence that is not treated subsequently. Mostly, these physicians are less skilled and do not have the opportunity to exchange information with colleagues, therefore specialized centres should be preferred. Our study analyses the value of surveillance imaging as part of the follow-up program. Our leading hypothesis is that many false positive diagnoses (benign) exist based on changes in surveillance imaging, implying that multiple operations and resulting physical and psychical damage can be avoided. Therefore, the aim of the study is to highlight the rate of false positives in the imaging follow-up of OSCC patients. Additionally, to analyse influence factors such as inflammation, scar tissue, contrast enhancement changes, and radiotherapy treatment to obtain a clear overview of the value of CT/NMRI as part of the overall follow-up process.

## 2. Material and Methods

### 2.1. Data

Patients with tumors with primary resection and safe margins with available follow-up data were included. Excluded were patients with tumors without primary surgery and a lack of data.

The patients were surgically treated in our university department of oral and maxillofacial surgery at Klinikum rechts der Isar Munich, Technical University Munich, and were included in our retrospective study. The OSCC classification is according to the latest Eighth TNM classification of the UICC. All these patients were regularly in the domestic follow-up routine program and had radiological or clinical radiological conspicuous findings. The study was conducted according to the Declaration of Helsinki, and the Ethical Committee of the Technical University Munich gave its full approval. The patients were segmented into two cohort groups: the clinical and radiological group = I and the radiological group = II. Group I included patients who had suspicious changes in surveillance imaging and were in the clinical follow-up. In contrast, group II comprised patients who had suspicious changes in surveillance imaging (CT/NMRI) and were clinically asymptomatic. The study focused only on CT and NMRI imaging because of their high relevance in the worldwide follow-up treatment of OSCC patients. The data comprised an amount of 125 patients, of which 56 appeared radiological and 69 appeared clinical and radiological conspicuous during their follow-up. The study included findings diagnosed from January 2017 to February 2020. The data contained doctors’ letters, radiological reports, and histological reports. All radiological reports were adjudged by the same experienced radiologist. The limitation of the study is a small sample size.

### 2.2. Statistical Analysis

A database with the information from the reports was constructed and coded accordingly. The database contained information such as patient age, gender, histological result, location of the biopsies (intraoral/extraoral), contrast enhancement changes, radiotherapy treatment, and findings of CT/NMRI. All those influence factors were set as independent variables, with the false positives as dependent variables. The database was coded as follows: malignant (1) and benign (2) split into scar tissue (3) and inflammation (4). Secondly, the data on radiotherapy was coded: patients who had no radiotherapy (0) and patients who underwent radiotherapy (1). Furthermore, the level of lymph node dissection as part of the extraoral biopsies was coded as level I (0), level II (1), level III (3), and level IV–V (4). The results were derived from chi-square tests, odds ratios (OR), and descriptive analyses with Excel Version 16.40. The significance level was set at *p* < 0.05.

## 3. Results

The results of the study are listed in the tables below. Table 1 shows general information on the study groups. Table 2 shows the histological results of the influence of radiotherapy. The distribution of the histological results regarding the locations of the biopsies is shown in Table 3, whereas Table 4 shows the results of the lymph node picking as part of the extraoral biopsies. The mean age was 63.6 years, most of the patients were male and had CT as surveillance imaging. Age was not included in statistical analyses because of the unimportance of our study’s aim. The distribution of the histological results in general as well as in both patient groups (I, II) was analysed. The chi-square test showed no significant dependence between the groups (*p* = 0.96) and the histological results, which implies that the type of cohort group is not significant for the findings of the biopsy. The result of the descriptive analyses was that 71 percent of the histological results were false positive (benign) findings in our study. Furthermore, the proportion of the histological results was analysed, being divided into malignant (1) and benign, which is split into scar tissue (3) and inflammation (4), to understand the origin of the false positive results. Therefore, the mode of the descriptive analyses was used, which shows us the most frequent histological result in our study. The most frequent mode is 4, which stands for inflammation. This suggests that most of the radiological results were mistaken in the case of inflammation.

In addition, the descriptive statistics showed that there were in total more patients with radiotherapy (*n* = 76, subgroup 1) than without (*n*= 49, subgroup 0). The two groups of findings were compared (I, II) to evaluate the frequency and influence of radiotherapy on the value of CT and NMRI. Table 2 depicts the effect of radiotherapy treatment on the quality of CT and NMRI. It compares the distribution of the histological results of patients in subgroups 0 and 1. Table 2 shows that there was a trend in both groups. The study showed more benign results, including inflammation and scar tissue, than malignant ones in CT follow-ups, regardless of the use of radiotherapy (*p* = 0.59). In contrast, the NMRI surveillance imaging showed as many benign as malignant results regarding cohort group II/subgroup 0 and only right positives (malignant) in cohort I/subgroup 0 (Table 2). Nevertheless, the same trend of false positives in CT imaging can be seen in NMRI imaging by patients who underwent radiotherapy treatment, too. The expression false positives means that suspicious results that were thought to be malignant were benign. The chi-square test showed no significant dependence between radiotherapy and the histological results (*p* = 0.59), which implies that experience and skills have a high value in evaluating surveillance imaging because changes in imaging are difficult to distinguish. The odds ratio showed an association between the subgroups 0 and 1 relating to contrast enhancement changes in NMRI surveillance imaging (OR = 5.14) as well as in CT imaging (OR = 1.39) but no significant effect (*p* = 0.11). These results imply that there was a higher disposition to contrast enhancement-induced artefacts due to radiotherapy in surveillance imaging. Additionally, the data showed an association between contrast enhancement and the histological result (malignant/benign) in NMRI imaging (OR = 1.80) but also in CT imaging (OR = 1.14). Hence, contrast enhancement had the tendency to show more positive results, though no statistical significance can be found (*p* = 0.39). Furthermore, the use of CT and NMRI showed a significant dependence (*p* = 0.03) on the histological result. The relevant data showed that there were greater odds of detecting malignant changes with NMRI than with CT imaging (OR = 2.62).

Another layer of results was derived from comparing intraoral and extraoral biopsies, including lymph node pickings (Table 4). The study revealed more intraoral biopsies than extraoral biopsies, as listed in Table 1. Also, the rate of lymph node picking as part of the extraoral biopsy is high (Table 1). Table 3 compares extraoral and intraoral biopsies divided into cohorts I and II. There were more benign results, including inflammation and scar tissue, in extraoral as well as intraoral biopsies (Table 3). The chi-square test showed a significant dependence between contrast enhancement changes in surveillance imaging and false positives (benign) in intraoral biopsies (*p* = 0.006). However, there was, in total, no significant dependence between the location of the biopsy (intraoral/extraoral) and the histological result neither in cohort I nor in II (*p* = 0.82).

To conclude, the data showed overall that contrast enhancement changes in surveillance imaging (CT/NMRI), which were more often in subgroup 1, the radiotherapy group, can lead to misinterpretation in radiological diagnosis. Above, follow-up with NMRI had greater odds of detecting right positive (malignant) findings.

## 4. Discussion

To our knowledge, it is the first study that compares CT and MRI in surveillance imaging with possible influence factors and consequences for diagnosis and treatment. Mostly, other studies analysed the imaging quality with PET/FDG and/or showed (dis-)advantages of CT and MRI. Furthermore, other studies show contrast enhancement changes and their positive effect on evaluating the DOI but not the influence on the quality of surveillance imaging and regarding, for example, only lymph node-positive patients [11,12]. Moreover, our study shows the outcome of surveillance imaging, especially regarding patients with asymptomatic recurrence, and the importance of detecting those recurrences early to guarantee the best treatment options which are required from other studies to guarantee the best treatment options and improve the cost effects for the health care system [13,14,15]. First, to mention the study of Pöpperl et al., this study evaluated PET CT in comparison to CT/MRI but only in the primary diagnosis and not in the aftercare. It is a special aspect that we evaluate radiology after therapies in the follow-up [14]. The evaluation results of PET CT are presented in the study of Scully et al., but not the findings of CT/MRI in aftercare [11]. Perez et al., in 2015, studied imaging diagnoses and concluded that there is still a lack of knowledge, especially in the aftercare of OSCC [15]. The advantages and disadvantages of CT/MRI in OSCC, in general, are shown by the study of Vishwanath et al. in 2020, but they do not include the histopathological results and the consequences of CT/MRI findings and they do not evaluate during the aftercare and they do not emphasize the role of recurrence without clinical appearance [12].

We see our study as a new scientific approach with importance and with new findings in OSCC therapy, especially aftercare treatment and recurrence diagnosis. Especially regarding patients with asymptomatic recurrence and the importance of detecting those recurrences early to guarantee the best treatment options.

The results of our study showed more false positive results (benign) similar in cohort I (*n* = 51) and II (*n* = 42) than right positive results (malignant). The results were mistaken, especially due to inflammation (Table 2) and radiotherapy treatment (Table 4). Patients who undergo radiotherapy treatment have to face several physical difficulties, such as mucositis, osteoradionecrosis, sensory disruption, and a high change in quality of life [16]. Especially regarding the head and neck area, patients with radiotherapy treatment have a higher risk for those physical and thus psychological changes [17]. As a consequence of radiotherapy changes, surveillance imaging quality can be limited [18]. Our study also showed a higher disposition for contrast enhancement-induced artefacts in surveillance imaging as a result of radiotherapy treatment. Bad imaging quality leads to difficulties in planning radiotherapy treatment [19] and in evaluating CT/NMRI for radiologists. Our study showed more false positives than malignant results due to inflammation, which is a significant irritation because of radiotherapy treatment [17]. Additionally, bad imaging quality can arise as part of artefacts like dental fillings, tooth crowns, and others [20]. Moreover, CT imaging is more prone to bad imaging quality caused by artefacts than NMRI [9], which could explain our results. Nevertheless, CT imaging is, among others, still popular because of its fast imaging and availability, so important decisions can be made more rapidly [8]. Physicians should individually consider which surveillance imaging method they would like to apply as structures are shown differently in CT and NMRI images. As we see it in our daily practice, it is also important that all follow-up images are completed with the same method as the primary method, so it is better comparable, and new findings are earlier and clearer to detect. In our point of view, MRI is helpful in some cases if there is the question of the extent of bone marrow infiltration, of artefacts of prosthodontics, small tumors of the palate, big tumors near the skull base, and the question of infiltration of relevant structures. High-risk patients should have a personalized follow-up protocol with individual periods of follow-up.

While CT has, for instance, better imaging for cortical erosion [21], NMRI is the better choice for muscle infiltration [9]. Thus, false positive results could be reduced by adapting the surveillance imaging method according to the situation. Others highlighted that more specificity can be achieved with combined imaging as DW-MRI with PET/CT [22] or follow-up with FDG-PET/CT [23]. Additionally, a study by Goerres et al. [24] implied that early detection can be achieved with PET, and following surgery, treatment may have a better chance of a cure. However, Rosenbaum et al. [25] underlined that the use of FDG-PET leads to artefact and false positives. FDG has no specificity for cancer and can also be seen in benign tissue with an inflammatory process [25]. Our study showed a high use of CT imaging and a high rate of false positive results. We found a significant dependence between the use of CT/NMRI and the histological result. The data implied that more positive results (malignant) can be found with NMRI than with CT imaging, which is one explanation for the study’s outcome. Another explanation could be that less experienced physicians have a disposition to see changes in imaging as suspicious changes. Consequently, there could be more false positives and more unnecessary operations, but fewer undetected recurrences. Moreover, it is described that surveillance imaging together with clinical follow-up is a good combination to detect early recurrence [2], thus reducing false positive results. Seventy-six percent of recurrences come up within the first two years after diagnosis, 11 percent within the third year, and only 61% of those cases are symptomatic [26]. However, our study showed no significant dependence of either cohort I or II on the histological results. Furthermore, one should consider if the physicians, in this case, especially the radiologists, have enough information about the patient’s disease and history, especially operative treatments in the past, to evaluate the imaging of CT/NMRI [27]. Furthermore, standardised data systems could also contribute to higher imaging quality by mitigating the effects of post-treatment changes in head and neck anatomy that radiologists have to face while assessing CT/NMRI [28]. Another study underlined these points by suggesting a more individual follow-up treatment, especially for patients with radiotherapy treatment. Their model recommends less frequent use of surveillance imaging methods, an interval of 7 to 9 months in the first three years, to improve treatment duration and costs for the health care system and the patients [29]. Overall, it is doubtful why CT imaging, or imaging in general, is still popular in OSCC follow-up treatment. There are several studies, though, that did not find any or only limited evidence on follow-up treatment and its positive effects on survival rates [30,31]. Ng et al. [29] underlined this fact by showing that there was no survival benefit with surveillance imaging, although recurrences were detected earlier. Roman et al. [32] indicated that physicians are primarily prone to using surveillance imaging because of the high perceived value, for instance, not missing recurrences, reassurance, and others. Moreover, other psychological factors seem to play an important role. On the one hand, patients are more convinced if they see no changes in their imaging results over time. On the other hand, a lot of physicians have a fear of missing out without imaging. Imaging provides physicians with a solid foundation to develop an opinion about the health status of a patient [32]. However, our study revealed that it is a challenge for physicians to evaluate changes in surveillance imaging. False positives in intraoral biopsies arising out of contrast enhancement changes as well as contrast enhancement changes due to tumor progress are difficult to distinguish. Therefore, specialized centers with experienced physicians should be preferred [7]. Even if early detection of recurrences can improve surgery treatment, as described above, a problem that physicians and patients must face are complications during and after surgery. Molecular pathological examinations of the tumor tissue (by RNA sequencing and immunohistochemistry), which are now increasingly becoming the focus of attention, could, in the future, provide an individual risk profile with regard to recurrence and lymph node metastases as early as the primary operation. These patients could then receive more frequent imaging in order to prevent recurrence at an earlier stage. The success of surgery treatment depends on many factors, for example, the tumor size, pre-surgery information, and the experience of the surgeon. Oncologic-certified centers with an experienced team of physicians can offer the best treatments [7,33]. There are intraoperative complications in patients who have already undergone surgery or radiotherapy, such as nerve damages and extensive bleeding due to fragile vessels. Postoperative complications could include, for instance, wound infection, wound disturbances, wound dehiscence, and respiratory insufficiency [34]. Unplanned reoperations as a consequence of wound dehiscence following protracted healing processes and a limited quality of life are a burden for patients, surgeons, and the health care system [35]. Confrontations with these complications are frustrating for patients as well as physicians; thus, operations must be chosen wisely.

## 5. Conclusions

In total, we need surveillance imaging for early detection, especially of asymptomatic recurrences. On the contrary, our study showed more false positive results due to misinterpretation of contrast enhancement changes, especially in intraoral biopsies and a lot of tissue changes due to inflammation. Thus, more personalized follow-up programs regarding special imaging should be considered. Moreover, the specific history of every patient should be observed at every clinical and radiological consultation to avoid misinterpretation as often as possible. A future tool to improve the evaluation of surveillance imaging could be machine learning. Due to increasing datasets in medicine, artificial intelligence acquires more information. Hence, the interpretation of imaging will be more precise and physicians will have an additional tool to reduce false positive results [36,37]. At least, operations should be chosen wisely to not harm the patient’s physiological and psychological condition, on the one hand, but also to see the need for early recurrence detection.

## Figures and Tables

**Table 1:** Distribution of the results.

Patient Group	*n*
I	69
II	56
>50 age	110
≤50 age	15
∅ age	63.3
male	78
female	47
CT	100
NMRI	25
extraoral	46
intraoral	79
suspicious lymph nodes	33
others from extraoral	13

*n* = number of patients, I = clinical and radiological group, II = radiological group.

**Table 2:** Distribution of the histological result of patients with and without radiotherapy treatment who had CT or NMRI during follow-up.

Patient Group	*n*			
	0		1	
	Malign	Benign	Malign	Benign
CT				
I	9	18	8	27
II	6	18	6	17
NMRI				
I	2	0	4	5
II	1	1	5	11

*n* = number of patients, I = clinical and radiological group, II = radiological group, 0 = no radiotherapy, 1 = radiotherapy.

**Table 3:** The location of the biopsies split into the histological result.

Patient Group	Malign	Benign	
		inflammation	scar tissue
	*n* = 36	*n* = 52	*n* = 45
I			
intraoral	14	22	14
extraoral	7	9	7
II			
intraoral	9	17	11
extraoral	6	4	13

*n* = number of patients, I = clinical and radiological group, II = radiological group.

**Table 4:** Suspicious lymph node levels during surveillance imaging and the outcome of the histological results.

Patient Group	Malign	Benign
I		
*n* = 25	*n* = 9	*n* = 16
level I (a + b)	2	4
level II (a + b)	2	5
level III	1	0
level IV + V	4	7
II		
*n* = 25	*n* = 9	*n* = 16
level I (a + b)	2	8
level II (a + b)	2	3
level III	3	0
level IV + V	2	5

*n* = number of patients, I = clinical and radiological group, II = radiological group.

## Data Availability

The data presented in this study are available on request from the corresponding author. The data are not publicly available due to ethical reasons.
